# Experimental and Theoretical Studies on the Adsorption Mechanisms of Uranium (VI) Ions on Chitosan

**DOI:** 10.3390/jfb9030049

**Published:** 2018-08-09

**Authors:** Kenji Mishima, Xiaoyu Du, Naoto Miyamoto, Naoki Kano, Hiroshi Imaizumi

**Affiliations:** 1 Department of Chemical System Engineering, Graduate School of Engineering, The University of Tokyo, Tokyo 113-8656, Japan; 2 Graduate School of Science and Technology, Niigata University, 8050 Ikarashi 2-Nocho, Nishi-ku, Niigata 950-2181, Japan; duxiaoyu90@163.com; 3 Department of Chemistry and Chemical Engineering, Faculty of Engineering, Niigata University, 8050 Ikarashi 2-Nocho, Nishi-ku, Niigata 950-2181, Japan; nmiyamoto@eng.niigata-u.ac.jp (N.M.); kano@eng.niigata-u.ac.jp (N.K.); analysis@eng.niigata-u.ac.jp (H.I.)

**Keywords:** chitosan, uranium (VI) ions, quantum chemistry calculation, pH dependence, adsorption, charges, molecular geometries

## Abstract

An experiment on the adsorption of uranium (VI) by chitosan was conducted to investigate the efficiency of chitosan as an adsorbent for U(VI). The adsorption potential of U(VI) by chitosan was investigated with ICP-MS by varying the experimental conditions such as the pH in order to obtain the optimum conditions. Adsorption dependence on the pH was confirmed, and the highest uptake of U(VI) was observed at pH 5. In addition, to scrutinize the experimental results, quantum chemistry calculations were performed. The results, taking into account the experimental conditions, show that the adsorption efficiency increases as the total charge of the adsorbent and adsorbate species decreases if both of them are positively charged. It was also found that a slight change in the adsorption geometric configuration controls the adsorption efficiency.

## 1. Introduction

It is well known that uranium (U) is one of the resources required to generate nuclear power and that it is ubiquitous in nature [1]. Although these features are advantages, it is very important to remember that U is a harmful pollutant in the natural environment. In particular, it has been pointed out that the mining and milling of U minerals generates a large amount of waste materials (tailings), which lead to environmental health damage [2,3,4,5,6,7]. To solve these problems, the proper management of U tailings by phytoremediation has been implemented recently [2,3]. This is because it is an environmentally friendly technology and uses plant biota to clean trace element contamination from soil [8]. To ensure ecosystem stability and public health, it is urgent to decrease the concentration of U(VI) to permissible limits in the environment [9].

On the other hand, due to the recent rapid advancement of computer facilities, it has become more and more realistic for quantum chemistry calculations to reproduce experimental results with sufficient accuracy. In fact, if we limit ourselves to the quantum chemistry calculations on U(VI) that were deemed to be difficult to deal with several years ago, we witness several numerical studies on uranium published so far, as follows.

For example, density functional theory (DFT), second-order Møller-Plesset perturbation (MP2), and coupled cluster singles + doubles (CCSD) calculations were used to calculate equilibrium structures, vibrational frequencies, and the nature of chemical bonds of hydrated UO_2_(OH)^+^, UO_2_(OH)_2_, NpO_2_(OH), and PuO_2_(OH)^+^ complexes in both first and second hydration spheres [10]. In addition, the structure of uranyl sorption complexes on gibbsites was studied by DFT and extended X-ray absorption fine structure (EXAFS) [11].

In relation to our interest, that is, quantum chemistry calculations on the adsorption of uranyl species on solid surfaces, several papers have been published so far.

For instance, using periodic boundary calculations and ab initio molecular dynamics simulations, it was predicted that hydroxylated titanium carbide (Ti_3_C_2_(OH)_2_) would be an efficient absorbent for uranyl ions in aqueous solutions, and several adsorption geometries and energetics were investigated [12].

In this work, however, our numerical interest is not concerned with any of the studies referred to above.

Our ultimate aim is to identify the factors controlling the adsorption efficiency that are consistent with both experimental and numerical results, and to propose a promising road map leading to highly efficient adsorption materials.

To approach this ultimate purpose, we set our experimental aim to investigate the efficiency of chitosan as an adsorbent for U(VI) and to show the future path for more practical and efficient use.

Among many biosorbents, chitosan can be an excellent biosorbent for metals because its amine (–NH_2_) and hydroxyl (–OH) groups may serve as coordination sites to form complexes with various heavy metal ions. In this study, chitosan (M.W. (molecular weight) 100,000–300,000) was purchased from ACROS ORGANICS Co. (Thermo Fisher Scientific, Waltham, MA, USA).

Specifically, the adsorption potential of U(VI) by chitosan was investigated with Inductively Coupled Plasma Mass Spectrometry (ICP-MS) (X series 2; Thermo Fisher Scientific) by varying the experimental conditions, such as pH, in order to obtain the optimum conditions. From these measurements, the adsorption dependence on the pH was confirmed. Furthermore, to evaluate the characteristics of the sample, the surface morphology of chitosan was determined by scanning electron microscopy (SEM) (Hitachi S-4300; Tokyo, Japan) and Fourier-transform infrared spectroscopy (FT-IR) (FTIE-4200; Jasco, Tokyo, Japan).

In addition, our theoretical aim was to pinpoint the factors dominating the adsorption efficiency on chitosan molecules that are not approached by experimental results alone. For this purpose, quantum chemistry calculations based on DFT were performed to calculate the stable geometries of the adsorbate-adsorbent complexes and compare the stabilization energies upon adsorption depending on the pH. From the calculation results, we found that the adsorption ability increases with a decrease in the electrostatic repulsion between the adsorbent and the adsorbate species, which is consistent with the experimental results. From a detailed examination of the numerical results, it was concluded that the most important factor dominating the adsorption capacity is the total charge numbers of the adsorbent and the adsorbate species, as well as their relative molecular geometries. Finally, it was found that U(VI) adsorbs on the surface of chitosan adsorbent much more strongly than chromium (VI) ions [13] do.

## 2. Experimental and Theoretical Details

### 2.1. Materials

**Figure 1:**
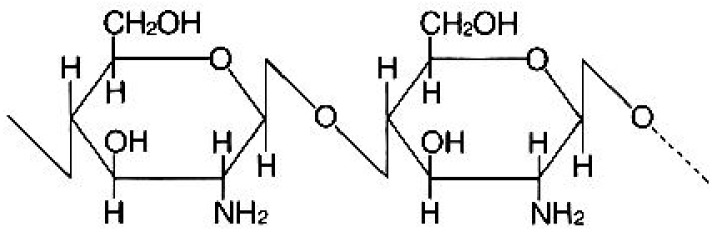
Molecular structure of chitosan.

Chitosan (molecular weight 100,000–300,000) was purchased from Acros Organics Co. (Morris, NJ, USA). The typical structure of chitosan is shown in Figure 1. Uranium (VI) standard solutions were prepared by diluting standard solutions (DWS-3; 20 mg·dm^−3^ 2% HNO_3_ solution) purchased from GL Sciences Inc. (Tokyo, Japan). All other chemical reagents were purchased from Kanto Chemical Co. Inc. (Tokyo, Japan). All reagents used were of analytical grade, and water (>18.2 MΩ) produced by an ultrapure water system (Advantec Aquarius, RFU 424TA, Tokyo, Japan) was employed throughout the work.

### 2.2. Adsorption Experiments

The adsorption of U(VI) from aqueous solution onto chitosan was investigated in a batch system. All adsorption experiments were performed in 100 mL flasks containing 50 mg chitosan and 50 mL aqueous U(VI) solution of the desired concentration. The pH value of the suspensions was adjusted by using NaOH and HNO_3_. The suspensions were shaken for 5 h by an automatic shaker (Plus shaker EP-1, TAITEC, Saitama, Japan). Subsequently, the suspensions were filtered, and then the filtrates were analyzed by ICP-MS to determine the U(VI) concentration. The adsorption study was carried out by varying the pH value (pH 1–8), contact time (30 min to 5 h), and adsorbent amount (0.2 g/L to 1.0 g/L). Adsorption isotherms of uranium (VI) onto chitosan were measured at varying initial U(VI) concentrations under optimized conditions.

### 2.3. Quantum Chemistry Calculations

All of the quantum chemistry calculations were performed using the Gaussian 09 suite of programs [14]. The reactants and the reaction intermediates were fully optimized with Becke’s three-parameter hybrid functional coupled with the Lee-Yang-Parr correlation functional (B3LYP). The basis sets were SDD (Stuttgart-Dresden) for uranium atoms and 6-311G (d,p) for H, C, N, and O atoms. To take into account the effect of the water environment on our target systems, the polarizable continuum medium (PCM method) with an appropriate dielectric constant of the solvent (water) was utilized [15].

The purpose of our calculations was to find the stable geometries of chitosan-uranium (VI) composites and to compare their relative energies. To do so, the following calculations were performed.

First, the uranium species ([UO_2_(H_2_O)_5_]^2+^ and [UO_2_(OH)(H_2_O)_4_]^+^) and chitosan dimers were fully optimized separately. The sum of energies of the uranium species and chitosan dimers are defined as the dissociation limit (the reactant).

Second, the systems composed of one uranium species and one chitosan dimer were partially optimized. In this case, the atoms found in the neighborhood of the sites of attachment of the uranium species and chitosan dimers were fully optimized, while those found farther away were frozen. The motivation for this partial optimization was to take into consideration the assumption that the chitosan would retain the original molecular structure while the adsorption process was occurring. Otherwise, the floppy structure of the chitosan dimer may have caused the molecule to modify it upon attachment of the uranium species, which may be unrealistic in the case of the chitosan polymer experiment.

Third, in order to apply the quantum chemistry calculations to chitosan, it is effectively impossible to take into account the long chain length of the chitosan polymer. One of the assumptions we made in the calculations was that it can be substituted with the chitosan dimer fully optimized beforehand.

Finally, it is realistic to study the adsorptions of [UO_2_(H_2_O)_5_]^2+^ and [(UO_2_)_2_(OH)_2_(H_2_O)_6_]^2+^ on chitosan dimers in the acidic region [14]. However, it is unrealistic to adsorb [UO_2_(H_2_O)_5_]^2+^ and [UO_2_(OH)(H_2_O)_4_]^+^ on chitosan dimers in the neutral region in view of the figure. The figure indicates that [(UO_2_)_3_(H_2_O)_5_]^+^ and [(UO_2_)_4_(H_2_O)_7_]^+^ are good candidates for studying the adsorption of uranyl ions in the neutral region. However, [(UO_2_)_2_(OH)_2_(H_2_O)_6_]^2+^, [(UO_2_)_3_(H_2_O)_5_]^+^, and [(UO_2_)_4_(H_2_O)_7_]^+^ are complicated and large, and may obscure the deduction of essential reaction mechanisms. Therefore, in order for the calculations to be self-contained, we assumed that it is possible to study the adsorption of [UO_2_(H_2_O)_5_]^2+^ and [UO_2_(OH)(H_2_O)_4_]^+^ on chitosan dimers in both the acidic and neutral regions.

## 3. Results and Discussion

### 3.1. Influence of pH on the Adsorption of U(VI)

**Figure 2:**
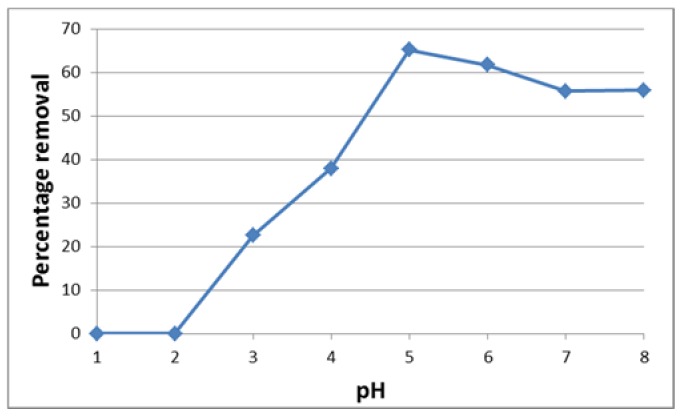
Effect of pH on U(VI) adsorption (time 5 h, adsorbent dose 1.0 g/L).

The adsorption of U(VI) on chitosan as a function of pH is shown in Figure 2. From this figure, it can be seen that the amount of U(VI) uptake increased by increasing the solution pH up to 5. The highest uptake was observed at pH 5, and uptake decreased slightly by increasing the pH from 5 to 8. It is known that U exists in different forms depending on the pH. U exists predominantly as a monomeric species, UO_2_^2+^, with small amounts of UO_2_ (OH)^+^ at pH ≤ 4.3, and at pH ≥ 5 colloidal or oligomeric species (i.e., (UO_2_)_2_(OH)_2_^2+^, (UO_2_)_3_(OH)_5_^+^, (UO_2_)_4_(OH)_7_^+^, (UO_2_)_3_(OH)_7_^−^) are formed [1,2,16,17].

That is, U usually exists in cationic species in solution at pH 5. Furthermore, from Figure 1, it can be seen that hydroxyl groups (–OH) are included in the chitosan.

From the above, it can be considered that the U(VI) adsorption occurs mainly by a cation exchange reaction between the H^+^ of hydroxyl groups on chitosan and cationic species of U(VI).

Apart from pH, we think that it is also important to include other effects such as the temperature and other co-existing anions on the adsorption efficiency of U(VI). Then it will be presented elsewhere in a future paper.

### 3.2. Adsorption Isotherms

**Figure 3:**
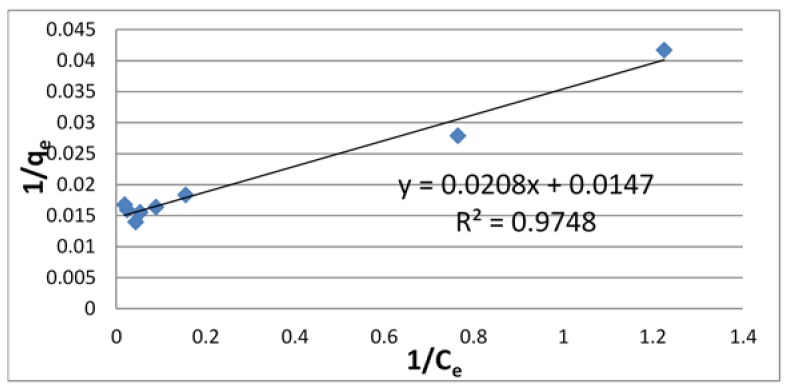
Langmuir adsorption isotherm of U using chitosan.

**Figure 4:**
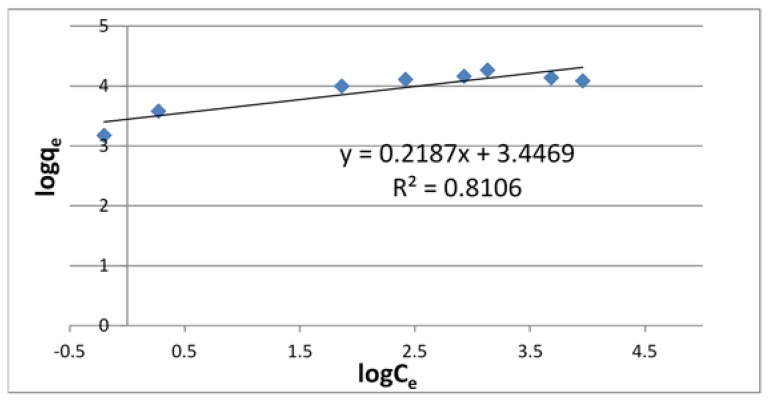
Freundlich adsorption isotherm of U using chitosan.

Adsorption isotherms are commonly used to reflect the performance of adsorbents in adsorption processes. In this paper, Langmuir and Freundlich isotherms were applied to the data obtained for U(VI) using chitosan. The linear plots of *C_e_*/*q_e_*-*C_e_* and lg*q_e_*-lg*C_e_* were presented for Langmuir (Figure 3) and Freundlich (Figure 4) models.

The correlation coefficient (*R*^2^) of these isotherms for U(VI) on chitosan is shown in Table 1 along with other relevant parameters. From this table, it is found that the *R*^2^ value is larger for the Langmuir isotherm than for the Freundlich isotherm. This result suggests that the adsorption on chitosan mainly occurred by monolayer reaction.

### 3.3. Kinetic Studies

**Figure 5:**
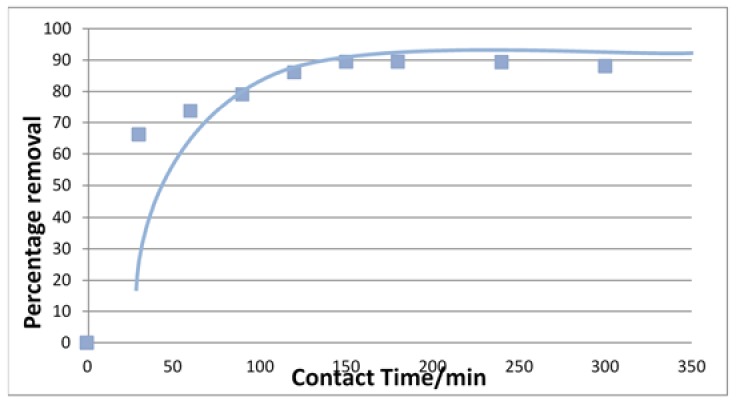
Effect of contact time on percent removal of U using chitosan.

The effect of contact time on U(VI) adsorption was investigated to study the adsorption rate of U(VI) removal. The percentage removal of U(VI) for the concentration of 20 μg/L with the adsorbent dosage 0.8 g/L at pH 5 are shown in Figure 5.

**Figure 6:**
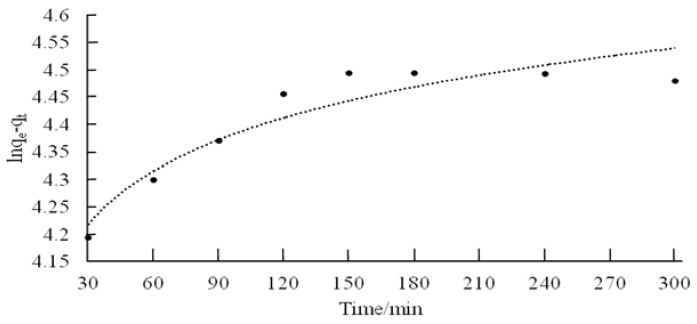
Pseudo-first-order kinetic fit for adsorption of chitosan.

**Figure 7:**
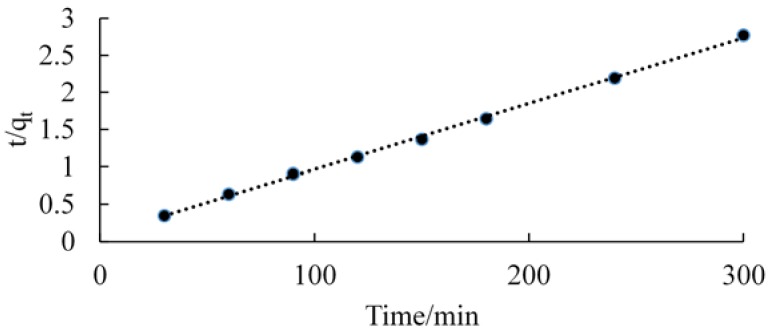
Pseudo-second-order kinetic fit for adsorption of chitosan.

The plots of ln(*q_e_* − *q_t_*) versus *t* for pseudo-first-order kinetics, and *t*/*q_t_* versus *t* for pseudo-second-order kinetics are shown in Figure 6 and Figure 7, respectively.

The kinetics data could be well described by pseudo-second-order plot. The observed experimental values for *q_e_*, and the values obtained from the plots of pseudo-first-order, and pseudo second-order kinetics are shown in Table 2. The *R*^2^ value of chitosan for pseudo-second-order rate kinetics is 0.999. Therefore, the rates of adsorption were found to conform to pseudo-second-order kinetic model.

### 3.4. Quantum Chemistry Calculations of the Adsorption of U(VI) on Chitosan

**Figure 8:**
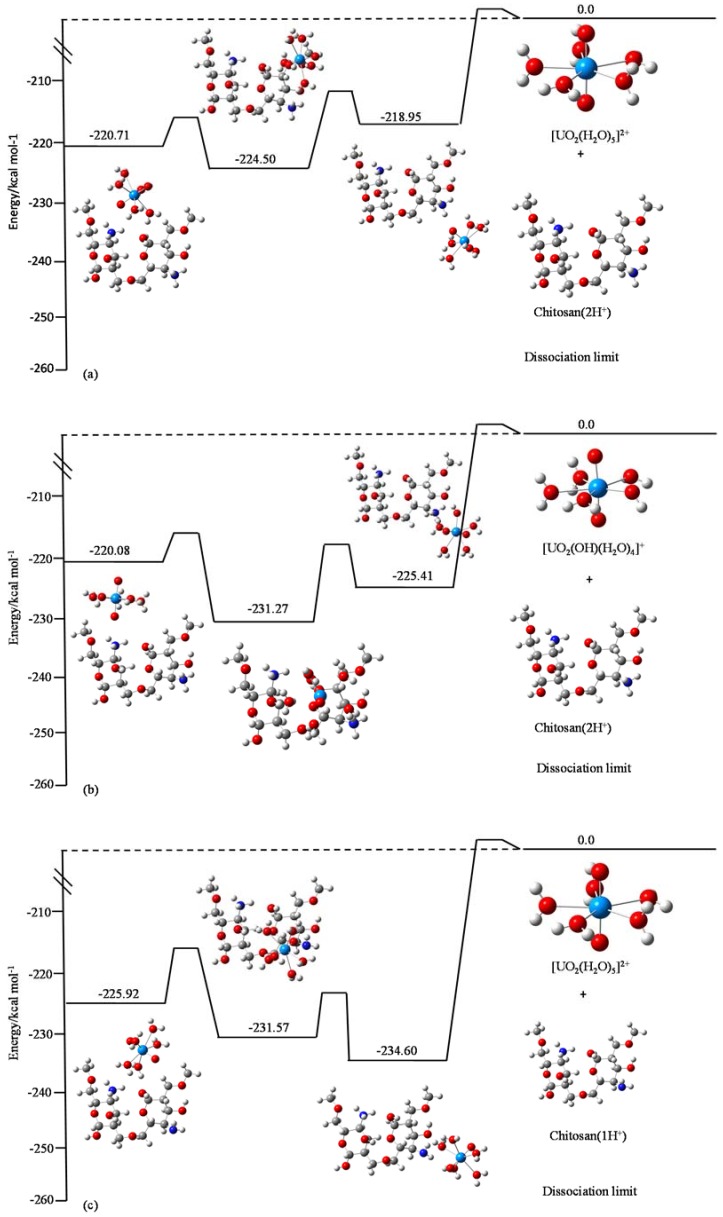

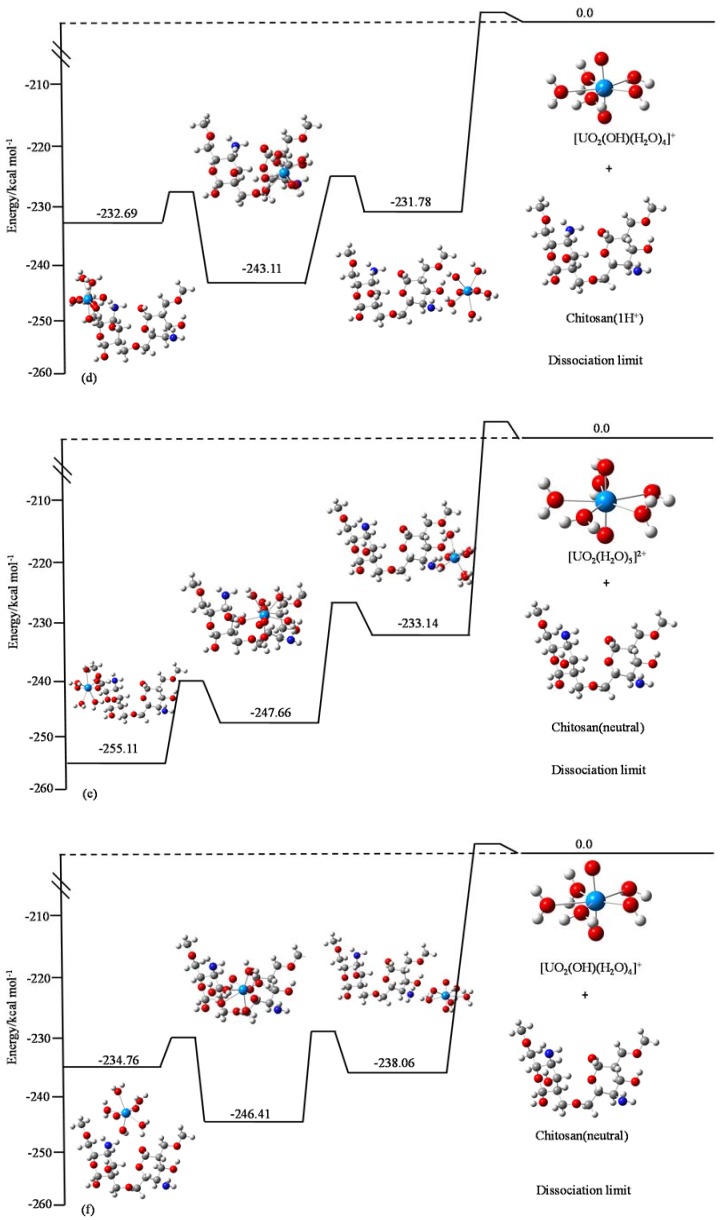
(**a**) [UO_2_(H_2_O)_5_]^2+^ and chitosan (2H^+^); (**b**) [UO_2_(OH)(H_2_O)_4_]^+^ and chitosan (2H^+^); (**c**) [UO_2_(H_2_O)_5_]^2+^ and chitosan (1H^+^); (**d**) [UO_2_(OH)(H_2_O)_4_]^+^ and chitosan (1H^+^); (**e**) [UO_2_(H_2_O)_5_]^2+^ and chitosan (neutral); and (**f**) [UO_2_(OH)(H_2_O)_4_]^+^ and chitosan (neutral). The term “chitosan (2H^+^)” means that two protons are attached to both nitrogen atoms of the chitosan dimer. The term “chitosan (1H^+^)” indicates that one proton is attached to one of the nitrogen atoms of the chitosan dimer. The term “chitosan (neutral)” stands for the neutral chitosan dimer. In the extremely low pH region, (**a**,**b**) are the most probable, (**c**,**d**) are the most probable in the pH region of around 3~6, and (**e**,**f**) correspond to the neutral region. The white, gray, blue, red, and light blue circles represent H, C, N, O, and U atoms, respectively.

It is considered that the distribution of uranium species varies depending on the pH (e.g., as shown in Figure 7 of [18]). Figure 8 shows the calculation results for six cases corresponding to three pH regions shown in Figure 7 of [18], with the calculation details shown in the caption.

From an inspection of Figure 8, the following conclusions can be drawn.

With the same chitosan dimer, the complex with [UO_2_(OH)(H_2_O)_4_]^+^ is more stable than the one with [UO_2_(H_2_O)_5_]^2+^ in the low pH regions (compare a with b, and c with d), except for Figure 3e,f. In Figure 3e,f, unlike the former case, the complex with [UO_2_(H_2_O)_5_]^2+^ is more stable than the one with [UO_2_(OH)(H_2_O)_4_]^+^. The former cases (Figure 3a–d) indicate that the adsorption complex becomes more unstable if the Coulomb repulsion between the adsorbent and the adsorbate increases. From this comparison, it can be concluded that the degree of difference of positive charges on the adsorbent and the adsorbate controls the adsorption efficiency.

On the other hand, the latter cases (Figure 3e,f) are contrary to our initial intuition. However, the tendency shown in these cases is, in fact, reasonable, because the Coulomb force does not work between the neutral adsorbent and the positively charged adsorbate. Therefore, if the adsorbent is neutral, factors other than the Coulomb force control the stability of the adsorbent-adsorbate complexes (i.e., the adsorption efficiency). It is found that uranyl ions are attached to the chitosan dimer in a face-on fashion in the most stable complex with stabilization energy −255.11 kcal/mol (Figure 3e) and the one with stabilization energy −246.41 kcal/mol (Figure 3f). In other cases (Figure 3e,f), the uranyl ions are attached in an edge-on fashion. This implies that the orbital hybridization between the adsorbent and the adsorbate plays an essential role in controlling the stabilization energy (i.e., the adsorption efficiency), in both the presence and absence of the Coulomb force. This conclusion is in accord with our previous study [13].

By combining the experimental and theoretical results, it was found that the most important factor controlling the adsorption efficiency of uranyl ions on dimer chitosan molecules is the difference of total charge numbers of the adsorbent and the adsorbate species and their relative molecular geometries.

In addition, because the room temperature (~300 K) corresponds to 0.60 kcal/mol, it is extremely difficult for all of the cases shown in Figure 8 to overcome the potential barrier to return to the dissociation limit (i.e., product). This means that the adsorption efficiency of chitosan is extremely good for uranyl ions in the whole pH region. This feature of uranium (VI) ions is one of the findings distinguishing them from chromium (VI) ions [13].

Finally, as shown by Equation (8) in [19], the change of potential energy, from the reactant to the product, is important for the discrimination between chemisorption and physical adsorption. Unfortunately, it is very difficult to calculate the potential energy from the experiment alone. Usually, it is in the range of several kcal/mol for the physical adsorption while it is in the range of 10~100 kcal/mol for the chemical adsorption. However, for some systems, the change of potential energy may deviate from these values greatly. Our system belongs to such a system, where the change of potential energy was in the range of 200 kcal/mol although all the reactions were physical adsorption. In this case, the best way will be the following. From the quantum chemistry calculations, one can easily calculate the potential energy with good accuracy and then using this value and other experimental values (e.g., pore radius) one can simulate the microspore capacity by the Dubinin-Radushkevich equation. By comparing the calculated results and those shown in the paper by the Dubinin-Radushkevich equation, one can finally conclude whether the adsorption is chemisorption or physical adsorption.

## 4. Conclusions

An experiment on the adsorption of uranium (VI) by chitosan was conducted to investigate the efficiency of chitosan as an adsorbent for U(VI). The effect of pH on the adsorption of U(VI) by chitosan was investigated, and the highest uptake of U(VI) was observed at pH 5.

In addition, the quantum chemistry calculations revealed that the total charge numbers of the adsorbent and the adsorbate and their relative molecular geometries are crucial in determining the adsorption efficiency.

Combining the experimental and theoretical results, we propose that one strategy to develop a new, highly efficient adsorbent-adsorbate combination is to maximize the difference between the total charge of the adsorbent and the adsorbate.

In the case of a pH smaller than 7, if the adsorbate is known to be positively charged in the liquid, it is better for the adsorbent to have as few hydroxy-, carboxyl-, or amino-groups attached by hydrogen ions as possible. This is because fewer hydrogen ions in the liquid are attached to these groups, which makes the adsorbent less positively charged. Therefore, the difference between the total charges of the adsorbent and adsorbate will be maximized. On the other hand, in the case of a pH larger than 7, the situation is quite different because there will be no well-known adsorbent negatively charged by the hydroxide ion. In this case, it may be better to choose or design the adsorbent and adsorbate that have the greatest difference of charges in the pH range larger than 7.

## Figures and Tables

**Table 1:** Coefficient of Langmuir and Freundlich isotherms for U(VI) using chitosan.

Langmuir Isotherm			Freundlich Isotherm		
*q*_max_	*K_L_*	*R* ^2^	*K_F_*	1/*n*	*R* ^2^
68.0	7.07 × 10^−2^	0.9748	31.4	0.219	0.8106

**Table 2:** Comparison between the adsorption rate constants, *q*_e_ and correlation coefficients associated with pseudo-first-order and pseudo-second-order rate equations.

Pseudo-First-Order Rate Equation			Pseudo-Second-Order Rate Equations		
*K*_1_ (min^−1^)	*q_e_* (mg/g)	*R* ^2^	*K*_2_ (min^−1^)	*q_e_* (mg/g)	*R* ^2^
0.140	2.121	0.887	0.288	2.127	0.999
